# Supplementary material for: Improvement in Muscle Fatty Acid Bioavailability and Volatile Flavor in Tilapia by Dietary α-Linolenic Acid Nutrition Strategy
Source: Foods. 2024 Mar 26;13(7):1005. doi: 10.3390/foods13071005 (PMC11011702; doi:10.3390/foods13071005)
Supplement: Supplementary file 1 [file foods-13-01005-s001.zip › foods-2882552-supplementary.pdf]

Table S1. Formulation and composition of the different experimental diets.

Diets	SO	BO
Formulation (%)		
Rapeseed meal	24	24
Soybean meal	40	40
Flour	16	16
Soybean oil[a]	9	/
Blend oil[a]	/	9
Microcrystalline cellulose	6.75	6.75
Monocalcium phosphate	2.0	2.0
Choline chloride	0.4	0.4
Premix[b]	1	1
L-methionine	0.35	0.35
Yeast extract	0.50	0.50
Proximate composition (%)		
Dry matter	90.78	91.00
Crude protein	32.35	33.41
Crude lipid	8.07	8.93
Crude ash	10.09	10.35
Main fatty acid composition (% total fatty acids)		
16:0	10.93	10.48
18:0	4.52	4.25
20:0	0.46	0.66
16:1	2.71	0.23
18:1	25.9	26.91
20:1	0.67	0.44
18:2n6 (LA)	55.45	42.85
18:3n3 (ALA)	6.55	13.56
LA/ALA	8.47	3.15

[a]Consists of soybean oil and linseed oil with proper proportions. Soybean oil contained 51.57% LA and 6.50% ALA, and linseed oil contained 16.3% LA and 47.44% ALA.

[b]Premix supplied the following minerals and/or vitamins (per kg of premix): Fe, 10 g; Zn, 3.2 g; Mn, 3 g; Co, 52 mg; iodine, 65 mg; Se, 15 mg; vitamin A, 3.3 mg; vitamin D_3, 0.08 mg; vitamin E, 307 mg; vitamin K_3, 1000 mg; vitamin B_1, 1500 mg; vitamin B_2, 2800 mg; vitamin B_6, 1000 mg; vitamin B_{12}, 8 mg; d-calcium pantothenate, 2000 mg; nicotinic acid, 7800 mg; biotin, 8 mg; folic acid, 400 mg; inositol, 12,800 mg; stable vitamin C, 20,000 mg.

Table S2 qPCR primers sequence used in this study.

Primer name	Primer sequence (5'-3')	Accession number
Lipid oxidation		
lox5-F	GTCTGGCAAATGTGAATTTGATCG	XM_003451927
lox5-R	CGATCAAATTCACATTTGCCAGAC	
lox12-F	CTGGTCAGTTAATGCCAGTTGC	XM_025903275
lox12-R	GGTGAGTGCGCAGCAGATGAGC	
lox15-F	GCACAGCAGAACACAACAGCGA	XM_025903464
lox15-R	GATTGTGAAGTGTATCTTAATTC	
gpx1-F	CCAAGAGAACTGCAAGAACGA	DQ355022.1
gpx1-R	CAGGACACGTCATTCCTACAC	
gpx4-F	CGCAGTGCCAAGTCCATCTATGA	GR617156.1
gpx4-R	GCATGGGAATCCCAGGATGCGT	
Lipid metabolism		
atgl-F	AAAACGTCCTGGTGACCCAGT	XM_003440346
atgl-R	TAGGAGGAATGATGCCACAGTAC	

	A	
lpl-F	CACCAAACTAGTGGGTCGTGATGT	NM_001279753.1
lpl-R	TCCCAGACTATAACCCAGCAGATGA	
dgat1-F	GCTTGAATTCTGTCACCCTGAAGA	XM_003444020.5
dgat1-R	ACCTGCTTGTAGGCGTCGTTCT	
dgat2-F	GCTTGAATTCTGTCACCCTGAAGA	XM_003458972.5
dgat2-R	ACCTGCTTGTAGGCGTCGTTCT	
fas-F	TCATCCAGCAGTTCACTGGCATT	GU433188.1
fas-R	TGATTAGGTCCACGGCCACA	
lpcat3-F	TTTTCGAGTGGTGGTCAAGG	XM_005455464.4
lpcat3-R	GCTACAACGCAGACAATCCC	
lpcat4-F	CTGGACAGGATGATTGACAGGT	XM_039610017
lpcat4-R	CACAGAGTGATCCTTGGAGT	
β actin-F	CAGGATGCAGAAGGAGATCACA	KJ126772.1
β atcin-R	CGATCCAGACGGAGTATTTACG	

Table S3 Fatty acid positional distribution of TAG and PL in the muscle of tilapia fed different diets.

Main fatty acid compositions (%)	SO		BO	
	sn-2	sn-1/3 or sn-1	sn-2	sn-1/3 or sn-1
TAG				
16:0	22.45 ± 0.35^{a}	17.76 ± 0.36^{b}	20.69 ± 0.63^{a}	15.24 ± 0.43^{b}
18:0	7.02 ± 0.46^{a}	3.10 ± 0.22^{b}	5.69 ± 0.38^{a}	2.83 ± 0.12^{b}
16:1	0.62 ± 0.15^{b}	1.48 ± 0.21^{a}	0.56 ± 0.10^{b}	1.32 ± 0.08^{a}
18:1	18.09 ± 0.44^{b}	25.20 ± 0.50^{a}	15.92 ± 0.42^{b}	23.75 ± 0.39^{a}
18:2n-6 (LA)	25.22 ± 0.31^{b}	33.22 ± 0.61^{a}	20.60 ± 0.53^{c}	24.19 ± 0.21^{b}
20:4n-6 (ARA)	0.49 ± 0.02^{b}	0.85 ± 0.06^{a}	0.31 ± 0.02^{b}	0.93 ± 0.11^{a}
18:3n-3 (ALA)	1.43 ± 0.08^{d}	4.55 ± 0.12^{c}	7.32 ± 0.06^{b}	13.36 ± 0.04^{a}
20:5n-3 (EPA)	0.44 ± 0.03^{c}	0.51 ± 0.04^{bc}	0.61 ± 0.04^{b}	0.88 ± 0.03^{a}
22:6n-3 (DHA)	6.96 ± 0.28^{b}	3.56 ± 0.14^{c}	9.75 ± 0.47^{a}	6.12 ± 0.14^{b}
SFA	33.26 ± 1.57^{a}	23.54 ± 0.48^{b}	29.85 ± 1.08^{a}	20.18 ± 0.61^{b}
MUFA	21.80 ± 0.52^{b}	29.38 ± 0.58^{a}	18.28 ± 0.54^{b}	27.15 ± 0.47^{a}
n-6 PUFA	29.08 ± 0.40^{b}	36.19 ± 0.77^{a}	24.18 ± 0.59^{c}	27.27 ± 0.76^{b}
n-3 PUFA	9.14 ± 1.31^{b}	10.95 ± 1.08^{b}	19.89 ± 1.48^{a}	20.69 ± 1.24^{a}
n-3 LC-PUFA	8.18 ± 0.30^{b}	4.70 ± 0.17^{c}	11.27 ± 0.50^{a}	7.88 ± 0.35^{b}
PL				

16:0	28.45 ± 1.33^{a}	20.19 ± 0.47^{b}	26.53 ± 2.05^{a}	18.02 ± 0.47^{b}
18:0	12.92 ± 0.12^{a}	6.04 ± 0.13^{b}	10.68 ± 0.27^{a}	5.24 ± 0.11^{b}
16:1	1.38 ± 0.07	1.45 ± 0.21	1.31 ± 0.07	1.55 ± 0.04
18:1	13.16 ± 0.44	15.63 ± 0.47	13.84 ± 1.81	15.08 ± 0.68
18:2n-6 (LA)	5.98 ± 0.10^{c}	17.66 ± 2.01^{a}	3.26 ± 0.07^{d}	11.10 ± 0.81^{b}
20:4n-6 (ARA)	6.97 ± 0.33^{a}	0.64 ± 0.07^{b}	6.22 ± 0.36^{a}	0.72 ± 0.18^{b}
18:3n-3 (ALA)	0.35 ± 0.02^{c}	1.33 ± 0.03^{bc}	2.52 ± 0.39^{b}	7.25 ± 0.04^{a}
20:5n-3 (EPA)	1.13 ± 0.01^{b}	0.27 ± 0.03^{c}	3.57 ± 0.11^{a}	0.33 ± 0.04^{c}
22:6n-3 (DHA)	14.49 ± 1.37^{b}	1.31 ± 0.02^{d}	19.96 ± 1.76^{a}	4.48 ± 0.03^{c}
SFA	43.59 ± 2.39^{a}	30.67 ± 1.83^{b}	40.54 ± 2.19^{a}	27.34 ± 1.65^{b}
MUFA	15.46 ± 1.78	20.43 ± 1.65	16.04 ± 1.76	19.51 ± 1.06
n-6 PUFA	13.87 ± 1.15^{c}	19.08 ± 2.44^{a}	10.80 ± 0.40^{d}	13.34 ± 1.65^{b}
n-3 PUFA	18.12 ± 1.58^{b}	4.59 ± 0.03^{d}	28.53 ± 1.55^{a}	12.44 ± 0.15^{c}
n-3 LC-PUFA	16.62 ± 2.37^{b}	2.08 ± 0.01^{d}	24.66 ± 1.45^{a}	6.01 ± 0.01^{c}

Notes: Values are means ± SE (n = 3) with nine fish per treatment. Values in the same row not sharing a common letter are significantly different ($p < 0.05$).

Table S4 Muscle volatile flavor compound contents of tilapia fed different diets

Main volatile flavor compound (%)	Dietary groups		Odor characteristics
	SO	BO	
Dimethyl-silanediol	0.12 ± 0.01^{a}	0.09 ± 0.00^{b}	Odorless
1-Octen-3-ol	0.03 ± 0.00	0.04 ± 0.00	Fishy, mushroom oily odor
1-Octanol	0.01 ± 0.00^{b}	0.03 ± 0.00^{a}	Oily odor
Ethyl ester-hexadecanoic acid	0.00 ± 0.00	0.00 ± 0.00	Waxy odor
Isopropyl myristate	0.00 ± 0.00^{b}	0.01 ± 0.00^{a}	Oily odor
5,9-Undecadien-2-one-6,10-dimethyl-(E)	0.01 ± 0.00^{b}	0.02 ± 0.00^{a}	Floral odor
Phosphonoacetic acid	0.01 ± 0.00	0.01 ± 0.00	Odorless
3-Methylsalicylic acid	0.01 ± 0.00	0.01 ± 0.00	Odorless
Decane	0.03 ± 0.00	0.03 ± 0.00	Fruity, sweet odor
4-Methyl-decane	0.01 ± 0.00^{a}	0.00 ± 0.00^{b}	Pungent acrid odor
2-Methyl-decane	0.01 ± 0.00^{a}	0.00 ± 0.00^{b}	Pungent acrid odor
3-Methyl-decane	0.02 ± 0.00^{a}	0.01 ± 0.00^{b}	Pungent acrid odor
Undecane	0.09 ± 0.00^{a}	0.07 ± 0.00^{b}	Faint odor
3,8-Dimethyl-decane	0.01 ± 0.00^{a}	0.00 ± 0.00^{b}	Gasoline-like odor
2,3,5-Trimethyl-decane	0.01 ± 0.00^{a}	0.00 ± 0.00^{b}	Gasoline-like odor
5-Methyl-undecane	0.01 ± 0.00^{a}	0.00 ± 0.00^{b}	Faint odor
3-Methyl-undecane	0.02 ± 0.00	0.02 ± 0.00	Faint odor
Dodecane	0.03 ± 0.00^{a}	0.02 ± 0.00	Oily odor
3-Methyl-tridecane	0.01 ± 0.00	0.01 ± 0.00	Gasoline-like odor
Tetradecane	0.01 ± 0.00	0.01 ± 0.00	Waxy odor
Pentadecane	0.01 ± 0.00	0.01 ± 0.00	Waxy odor
Hexadecane	0.01 ± 0.00^{a}	0.00 ± 0.00^{b}	Waxy odor
Heptadecane	0.01 ± 0.00	0.01 ± 0.00	Odorless
2,6,10,14-Tetramethyl-hexadecane	0.01 ± 0.00^{a}	0.00 ± 0.00^{b}	Odorless
Nonanal	0.10 ± 0.00^{b}	0.20 ± 0.00^{a}	Oily, floral and fruity odor

Decanal	0.04 ± 0.00[b]	0.08 ± 0.00[a]	Oily, waxy and fruity odor
Dodecanal	0.01 ± 0.00[b]	0.02 ± 0.00[a]	Floral and oily odor
Tetradecanal	0.00 ± 0.00[b]	0.01 ± 0.00[a]	Oily, waxy, fishy and fruity odor
Pentadecanal	0.01 ± 0.00	0.02 ± 0.00[a]	Sweet odor
Octadecanal	0.00 ± 0.00	0.00 ± 0.00	Fruity, fishy and herbal odor
2,4-Decadienal	0.00 ± 0.00	0.01 ± 0.00[a]	Oily odor
N,N-dibutyl-formamide	0.02 ± 0.00[b]	0.04 ± 0.00[a]	Fishy odor
Butylated hydroxytoluene	0.02 ± 0.00[a]	0.01 ± 0.00	Odorless

Notes: Values are mean ± SE (n = 3) with nine fish per treatment. Values in the same row not sharing a common letter are significantly different ($p < 0.05$). Odor characteristics are described according to the literature (Cai et al., 2021; Jones et al., 2022).
